# Impact of thermal stress on placental function and fetal physiology

**DOI:** 10.21451/1984-3143-AR2018-0056

**Published:** 2018-08-03

**Authors:** Sean W. Limesand, Leticia E. Camacho, Amy C. Kelly, Andrew T. Antolic

**Affiliations:** School of Animal and Comparative Biomedical Sciences, University of Arizona, Tucson AZ 85719 USA

**Keywords:** heat stress, intrauterine growth restriction, placental insufficiency, sheep fetus.

## Abstract

In ruminants, prolonged exposure to high ambient temperatures negatively affects placental development and function. The pursuing limitations in placental oxygen and nutrient supply between the mother and fetus slow fetal growth lowering birth weights and postnatal performance. The pregnant ewe is a long-standing animal model for the study of maternal- fetal interactions and is susceptible to naturally occurring heat stress, which causes fetal growth restriction. In the pregnant ewe, studies show that the fetus adapts to hyperthermia-induced placental insufficiency to preserve placental transport capacity of oxygen and nutrients. These adaptive responses are at the expense of normal fetal development and growth. Enlarged transplacental gradient for oxygen and glucose facilitates diffusion across the placenta, but develops by lowering fetal blood oxygen and glucose concentrations. Fetal hypoxemia and hypoglycemia slow growth and alter their metabolic and endocrine profiles. Deficits in amino acids transport across the placenta are present but are overcome by reduced fetal clearance rates, likely due to fetal hypoxemia or endocrine responses to hypoxic stress. Here, we provide an overview of the performance limitations observed in ruminants exposed to heat stress during pregnancy, but we focus our presentation on the sheep fetus in pregnancies complicated by hyperthermia-induced placental insufficiency. We define the characteristics of placental dysfunction observed in the fetus of heat stressed ewes during pregnancy and present developmental adaptations in organogenesis, metabolism, and endocrinology that are proposed to establish maladaptive situations reaching far beyond the perinatal period.

## Introduction

Environmental heat stress diminishes revenue for livestock producers by negatively impacting nutrient utilization, growth, and reproductive performance. Consequences of heat stress on early embryonic survival have been well documented, both scientifically and economically (Hansen *et al*., 2001). However, financial losses from warm environmental conditions are not limited to embryonic wastage. Maternal exposure to prolonged high ambient temperatures during gestation has been associated with lighter birth weights, greater incidence of morbidity before weaning, lower survival rates, and less desirable carcass traits (Shelton, 1964; Monteiro *et al*., 2016). As we will explain, these latter complications likely are products of developmental adaptations to nutrient and oxygen deprivation caused by hyperthermia-induced placental insufficiency. During maternal heat stress, fetal growth restriction may be considered beneficial for the dam, as a smaller conceptus yields less metabolically active tissue, greater maternal surface area to mass ratio, and less nutritional strain on the mother (Wells and Cole, 2002). Although fetal growth restriction is advantageous for the dam, fetal growth restriction and the accompanying adaptations to placental restriction are associated with a myriad of metabolic complications that negatively affect future performance.

Because offspring from a heat stressed dam are growth restricted during gestation, we begin with a closer look at the characteristics of placental dysfunction that restrict fetal growth and cause metabolic adaptations. We provide an overview of the performance limitations observed with maternal heat stress in ruminants, but focus our presentation on work conducted in sheep that are experimentally heat stressed during mid gestation, a time when the placenta is established and placental growth is at maximum (Regnault *et al*., 2002a). The pregnant sheep has been used extensively over the past 50 years to investigate placental and fetal physiology due to the ability to surgically place and maintain catheters in the maternal and fetal vasculature that allow for repetitive blood sampling from non-anesthetized ewes (Meschia *et al*., 1965; Barry *et al*., 2008). The substantial groundwork on maternal-fetal interactions in sheep provides ample knowledge for normal pregnancy, as well as information on models of pregnancies complicated by experimentally or naturally produced placental restriction, which includes a model of hyperthermia- induced placental insufficiency.

**Figure 1:**
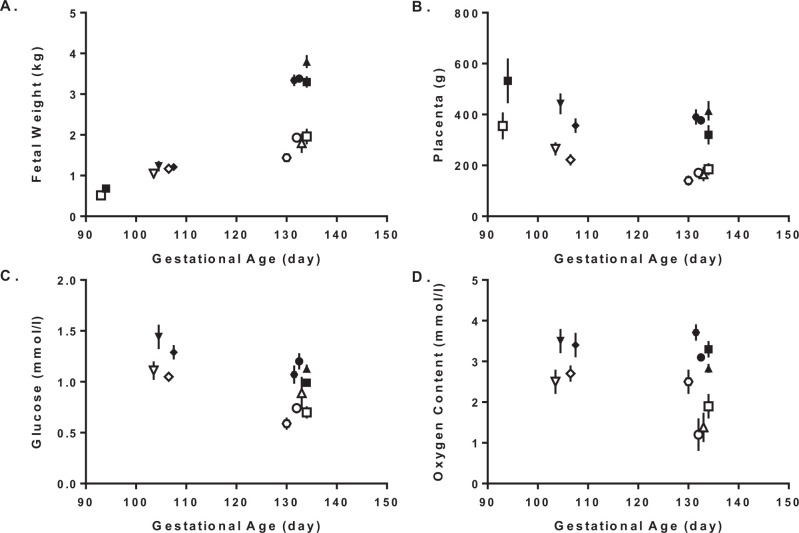
Progression of hyperthermia-induced placental insufficiency. Fetal weights (A), placental weights (B), fetal arterial plasma glucose concentrations (C), and fetal arterial blood oxygen contents (D) are presented as means reported in Ross *et al*., 1996 (hexagon); Brown *et al*., 2012 (circle); de Vrijer *et al*., 2006 (square <100 days); Limesand *et al*., 2013 (downward triangle), Macko *et al*., 2013 (diamond); and Rozance *et al*., 2018 (square >130 days). Each individual point represents a group mean for thermoneutral control ewes (fill shapes) and heat stress ewes (open shapes) for that specific report (symbol). At younger gestational ages (<110 days), all group means were significantly different within a report, except for fetal weight. At older gestational ages (>130 days), group means within the report were significantly different. Conclusions from these data indicate that advancing gestational age leads to greater differences in all parameters measured, which includes slower rates of fetal growth.

Pregnant ewes exposed chronically to high ambient temperatures in the laboratory from early to late gestation have fetuses that are significantly growth restricted close to term (Bell *et al*., 1987; Thureen *et al*., 1992). Ultrasonographic measurements indicate that biometric parameters for determining fetal growth restriction, for example abdominal circumference begins to diverge from normal as early as mid-gestation. This is a developmental point, prior to rapid fetal growth and at the apex of placental growth (Galan *et al*., 1999). Terminal studies indicate that significant reduction of placental mass precedes declines in fetal weight (Fig. 1A and B). Before 110 days of gestation (term 149 days), placental weights were significantly less in heat stress ewes than controls (280 ± 32 g vs. 443 ± 32 g).

However, fetal weights were not affect at this younger age (0.9 ± 0.2 kg versus 1.0 ± 0.2 kg). After 130 days of gestation, both fetal and placental weights were significantly less in heat stressed ewes compared to controls (49% for fetus and 56% for placenta). These data indicate that the majority of fetal growth restriction occurs during the final stages of gestation after placental growth restriction (de Vrijer *et al*., 2006; Macko *et al*., 2013). Limitations are caused by reduced placental mass and function, which leads to the development and progressive decline in fetal glucose (21 to 33% less) and oxygen (25 to 46%) concentrations over the final third of gestation, when fetal growth rate is at maximum (Limesand *et al*., 2013; Rozance *et al*., 2018; Fig. 1C and D). This evidence supports the hypothesis that placental deficiencies are responsible for fetal growth restriction in late gestation. Abnormal placental growth, vascular organization, and angiogenesis were described as possible causes of placental insufficiency due to aberrant expression patterns of angiogenic growth factors and their receptors (Vatnick *et al*., 1991; Regnault *et al*., 2002b; Galan *et al*., 2005; Hagen *et al*., 2005). Together, the pregnant ewe and this model of hyperthermia-induced placental insufficiency provides a unique opportunity to investigate fetal adaptive responses and growth restriction caused by a naturally induced placental restriction, which negatively effects their future health and performance. We review the outcomes in the placenta and fetus that are associated with adaptive responses to hyperthermia-induced placental insufficiency and discuss how they relate to future deficiencies in production.

## Effects of heat stress in ruminants during gestation and lactation

Environmental heat stress imposes significant limitations on fetal growth and milk production in several species of ruminants, but some species, usually those with higher production rates, are more susceptible to heat stress. For this reason, lactating dairy cows have a low tolerance to heat stress because as milk production per cow is increased there is a concurrent increase in metabolic heat production (Collier *et al*., 2006). Similarly, substantial economic consequences are incurred during gestation and lactation due to high metabolic heat output (West, 2003; Collier *et al*., 2006).

Mechanisms that explain placental and fetal growth restriction include the redistribution of blood flow to the skin and nasal mucosa at the expense of other internal organs including the pregnant uterus (Reynolds *et al*., 1985; McCrabb *et al*., 1993). Reductions in placental function, along with maternal factors caused by heat stress, restrict mammary gland development and lower the potential yield for the subsequent lactation. In addition to losses in milk yield and quality following the immediate pregnancy, heat stress in late pregnancy effects immune processes associated with poor transition to lactation including: lower phagocytic activity and decreased hepatic prolactin signaling (PRL- R, SOCS-3, and CAV-1 mRNA) during the dry period (do Amaral *et al*., 2011). Milk yield and milk protein are influenced by calving season, as both are lowest during the warmest months compared to cows that calved in the winter (Barash *et al*., 2001). Lactating sheep are also susceptible to heat stress, which leads to differences in milk composition, specifically lower fat and protein content (Abdalla *et al*., 1993). In addition to lighter birth weights, calves from heat stress cows exhibited both immediate and prolonged effects on their passive immunity, growth, activity patterns, and thermotolerance (Ahmed *et al*., 2017; Laporta *et al*., 2017). Furthermore, the effects of heat stress on offspring persist, resulting in lower yearling weights and less heifers reaching their first lactation. Cows exposed to heat stress during fetal life produced less milk compared to cows that received heat abatement strategies (Monteiro *et al*., 2016). This evidence shows that heat stress during gestation affects fetal development and also creates lasting complications that lower the future productivity of the offspring, which may be caused by developmental adaptations to placental insufficiency.

## Maternal heat stress limits placental transport capacity

The capacity of the placenta to transfer oxygen and nutrients must increase throughout pregnancy to meet metabolic demands of the growing fetus. In sheep, placental transport capacity continues to increase by expanding the surface area of the maternal-fetal interface and by thinning of the placental barrier to promote the exchange and permeability of metabolic substrates. Amino acids, oxygen, and glucose are transported across the placenta by active transport, passive diffusion, and facilitated diffusion (Battaglia and Meschia, 1978). For diffusion mechanisms, the rate of transplacental transport is dependent on uterine and umbilical blood flow, substrate permeability, and substrate concentration difference across the placenta.

**Figure 2:**
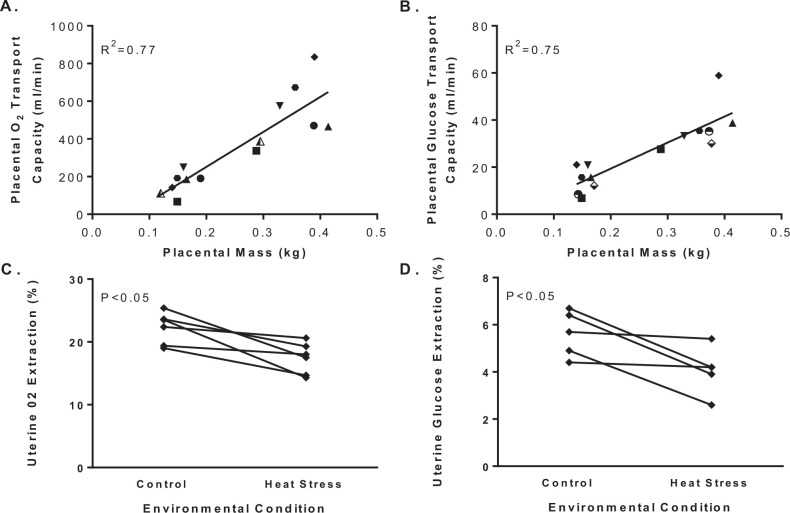
Placental transport capacity and uterine extraction efficiency for oxygen and glucose. Placental transport capacity for oxygen (A) and glucose (B) are presented by placental weights for independent group means from previously reported cohorts of thermoneutral and heat stressed ewes during gestation. Placental transport capacity is the net umbilical (fetal) uptake (µmol/min) divided by the maternal arterial-fetal arterial plasma concentration difference (µmol/ml) according to the equation: transport capacity = uptake/concentration difference. Linear regression analysis shows a positive association for placental transport capacity and placental mass (R2). Uterine extraction of oxygen was calculated by expressing the whole blood arterial-venous oxygen concentration difference across the uterine circulation as a percent of the arterial concentration (C). Uterine extraction of glucose was expressed as the plasma arterial-venous concentration difference as a percent of the arterial glucose concentration (D). Group means for thermoneutral control (fill symbols) and heat stressed animals (open symbols) were reported in Bell *et al*., 1987 (downward triangle); Thureen *et al*., 1992 (hexagon); Ross *et al*., 1996 (diamond); Anderson *et al*., 1997 (small circle, panel A); Regnault *et al*., 2003 (large circle, panel A); Limesand *et al*., 2004 (circle, panel B); de Vrijer *et al*., 2004 (triangle); Limesand *et al*., 2007 (large square, panel B) and Brown *et al*., 2012 (small square, panel B). An ANOVA with reported study as the repeated measure identify group (control and heat stress) differences (P-values) for panels C and D.

**Figure 3:**
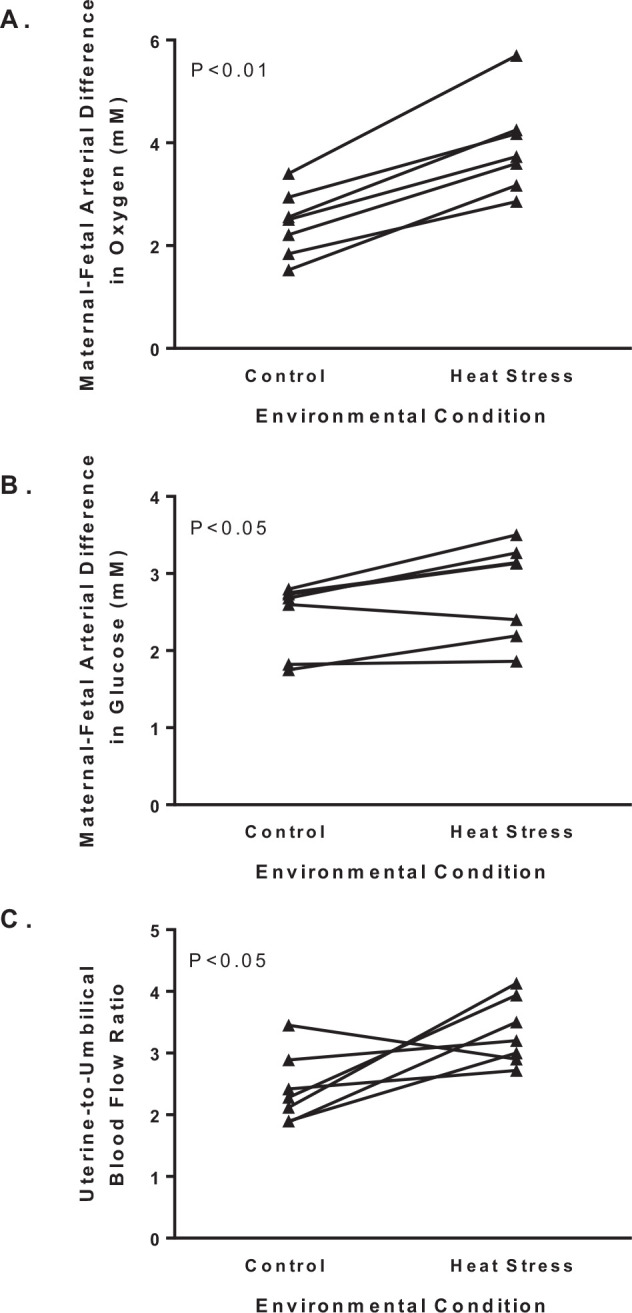
Transplacental gradients and uterine-umbilical blood flow ratio. Maternal- fetal arterial difference for oxygen (A) and glucose (B) concentrations are presented for pregnant ewes at approximately 90% of gestation. Uterine-to-umbilical blood flow rates are presented in panel C. Group means for thermoneutral control and heat stressed ewes were reported in Bell *et al*., 1987; Thureen *et al*., 1992; Ross *et al*., 1996; Anderson *et al*., 1997; Regnault *et al*., 2003; Limesand *et al*., 2004, 2007; de Vrijer *et al*., 2004. An ANOVA with report as the repeated measure identifies group (control and heat stress) differences (P-values).

Placental clearance is diminished with heat stress due to lower permeability for metabolic substrates. Oxygen and glucose permeability is reduced by a smaller placenta with less surface area and transport capacity, which combine to lower uterine extraction efficiency (Fig. 2). Evidence for this conclusion is that the transplacental gradients of oxygen and glucose increase in ewes exposed to environmental heat stress during pregnancy compared to pregnant ewes maintained under thermoneutral conditions (Fig. 3). Unlike placental transfer rates, placental clearance is independent of concentration gradients, but dependent on the properties of the exchanger (membrane) permeability or perfusion. Studies with inert molecules that have flow-limited placental transport show equivalent transplacental clearance rates in heat stressed and thermoneutral ewes. For example, there is no difference in ethanol clearance across the placenta between thermoneutral and heat stressed ewes when expressed relative to placental mass (Bell *et al*., 1987; Thureen *et al*., 1992; Regnault *et al*., 2007). This observation excludes shunting or uneven perfusion of uterine and umbilical blood flow as a cause for decreased transplacental clearance in heat stressed ewes. In addition to lower placental permeability, placental transport capacity of metabolic substrates is hindered by alterations in uteroplacental consumption of the substrate itself. In heat stressed ewes, uteroplacental oxygen consumption normalized to placental weight is unaffected, and glucose utilization by uteroplacental tissue is less (Bell *et al*., 1987; Thureen *et al*., 1992; Regnault *et al*., 2007). Therefore, the enlargement in the transplacental concentration difference for oxygen and glucose are due to lower placental permeability, which for glucose may be explained partially by lower abundance of facilitated glucose transporters (Limesand *et al*., 2004; Wallace *et al*., 2005).

**Figure 4:**
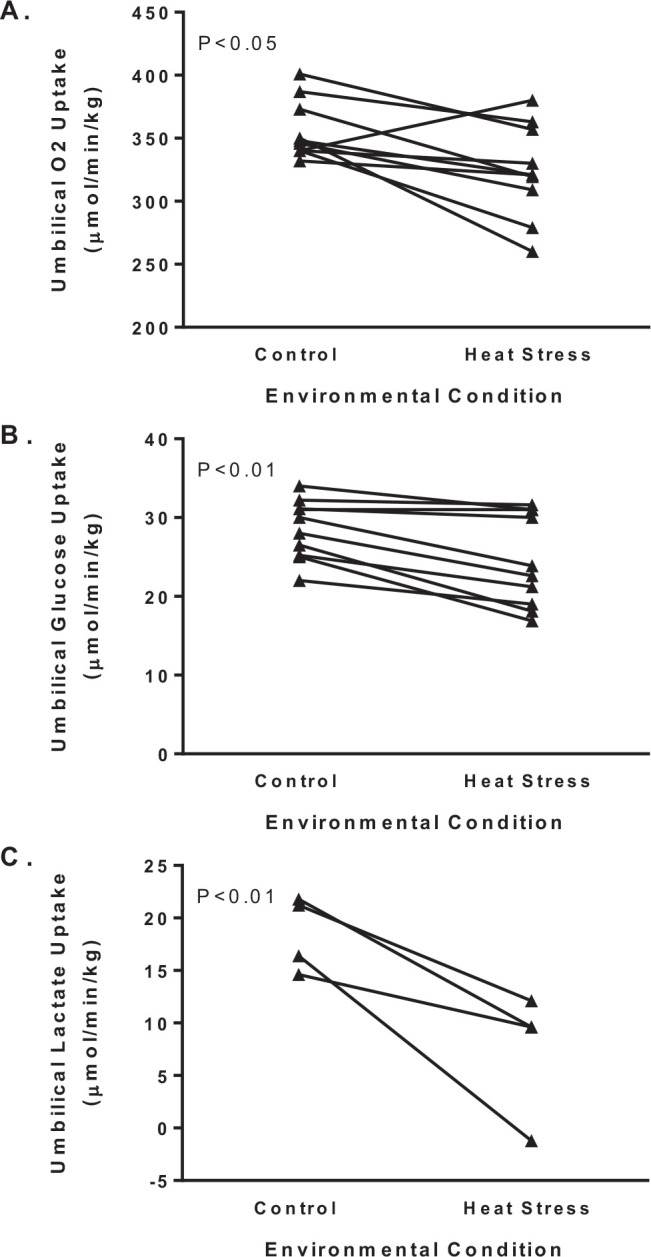
Umbilical uptakes for oxygen, glucose, and lactate. Fetal weight-specific net umbilical uptakes for oxygen (A) and glucose (B) are presented for pregnant ewes at 90% of gestation. Group means for thermoneutral control and heat stressed ewes were reported in Bell *et al*., 1987; Thureen *et al*., 1992; Ross *et al*., 1996; Anderson *et al*., 1997; Regnault *et al*., 2003; Limesand *et al*., 2004, 2007; de Vrijer *et al*., 2004; Brown *et al*., 2012, 2015; and Thorn *et al*., 2013. An ANOVA with reported study as the repeated measure identifies group (control and heat stress) differences (P-values) for each of the uptakes.

Transplacental gradients and uterine-umbilical blood flow ratios adapt under heat stress to preserve the net umbilical uptake of glucose and oxygen, but as discussed later, this compensatory mechanism causes reductions in substrate concentrations in the fetus that become detrimental to development and growth (Bell *et al*., 1987; Thureen *et al*., 1992; Regnault *et al*., 2007, 2013). Simple concepts for diffusion indicate that larger transplacental concentration gradients (Fig. 3A and B) will increase the net movement of oxygen and glucose across the placenta to the fetus. Another compensatory mechanism is greater uterine-to-umbilical blood flow ratio, which further demonstrates impaired placental diffusion capacity in heat stressed ewes (Fig. 3C). Normally the uterine-to-umbilical blood flow ratio is ~2, which is predicted to fulfill the delivery requirements for the placenta and fetus because maximum fetal clearance occurs when flows are equivalent (Wilkening *et al*., 1982). Increases in the blood flow ratio lowers uterine arteriovenous differences for oxygen and aids in expanding the transplacental gradient to facilitate uterine uptake (Bell *et al*., 1987; Regnault *et al*., 2003). This adaptation may be advantageous to the fetus because it lowers cardiac output to the placenta to increase umbilical uptake, but the increase vascular resistance in the placenta is postulated to negatively affect the cardiovascular development (Galan *et al*., 2005). Together, the enlarged transplacental concentration gradients of oxygen and glucose and increased uterine-to-umbilical blood flow ratio are sufficient to minimize reductions in net umbilical oxygen and glucose uptake per fetal mass. However, comparisons for means across several reported cohorts indicate that there were modest but significant reductions of 9 and 14% in net umbilical oxygen and glucose uptakes, respectively (Fig. 4).

Placenta delivery of amino acids to the fetus is also lower in heat stressed ewes even though fetal plasma amino acid concentrations are not necessarily reduced (Thorn *et al*., 2009; Regnault *et al*., 2013). For most amino acids, concentrations in fetal circulation are normally greater than in maternal circulation and therefore are transported actively across the placenta against their concentration gradient. In heat stressed ewes, the absolute placental flux for essential amino acids from mother to fetus is reduced ~80%, whereas the flux relative to placental mass is ~40% less (Ross *et al*., 1996; Anderson *et al*., 1997; de Vrijer *et al*., 2004). Similar reductions in transplacental uptake of essential amino acids are seen when expressed relative to fetal mass, with the exception of lysine (Regnault *et al*., 2013). Two important points become evident from these independent studies on amino acid placental transport in heat stress ewes. First, fetal uptakes of essential amino acids are reduced to similar magnitudes. Second, the impaired transport of amino acids is due to a reduction in placental size as well as decreased transport capacity per unit mass. Therefore, amino acid transfer depends on surface area of the maternal-fetal interface, which is reduced, as well as on the concentrations of amino acid transporters (Regnault *et al*., 2005). Interestingly, decreased transplacental flux of amino acid does not always lower their concentration in fetal plasma, implicating adaptive mechanisms in fetal metabolism or clearance of amino acids, which were associated with low oxygen concentrations (Regnault *et al*., 2013). Again, these data show that placental insufficiencies produced by heat stress depend on decreased placental mass and function, even though compensatory mechanism by the fetus are in place to assist with the placental deficiencies.

## Fetal responses to hyperthermia-induced placental insufficiency

**Figure 5:**
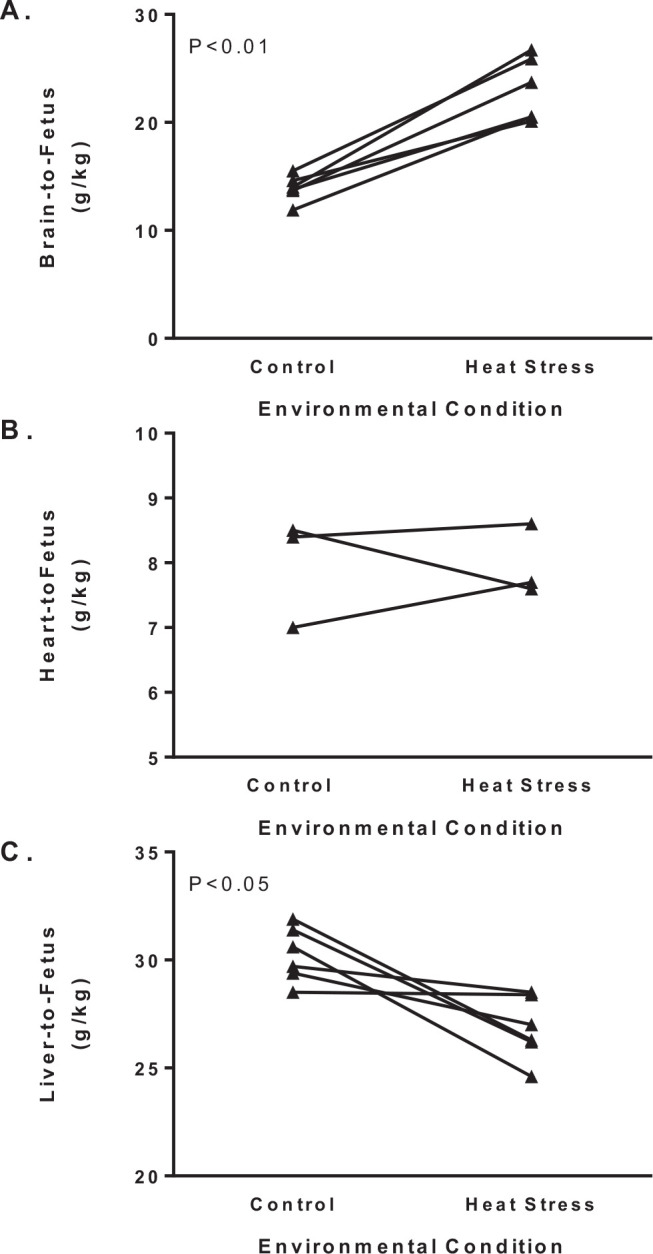
Asymmetric growth restriction with hyperthermia-induced placental insufficiency. Brain (A), heart (B), and liver (C) weights in grams are expressed relative to fetal weight (kg) for fetuses necropsied at 90% of gestation. Group means for thermoneutral control and heat stressed ewes were reported in Thureen *et al*., 1992; Anderson *et al*., 1997; de Vrijer *et al*., 2004; Brown *et al*., 2012; Davis *et al*., 2015; and Barry *et al*., 2016. An ANOVA with reported study as the repeated measure identifies group (control and heat stress) differences (P-values) for organ to fetal weight ratios.

Maternal hyperthermia is natural in sheep that, uncharacteristically, carry pregnancies in summer months producing a smaller placenta with lower transport capacity for glucose, amino acids, and oxygen. Therefore, hyperthermia-induced placental insufficiency under-nourishes the fetus and leads to asymmetric intrauterine growth restriction (IUGR) that spares brain and heart growth relative to overall body weight (Fig. 5). In heat stressed sheep with placental insufficiency, we and others have characterized fetal adaptations in metabolism, endocrinology, and selected organ functions to specify mechanisms responsible for developmental programming. These studies have focused on fetal metabolism and endocrinology, pancreatic insulin secretion, hepatic glucose production, skeletal muscle growth and metabolism, and cardiac metabolism. In combination with oxygen and nutrient deficits, elevations in norepinephrine and epinephrine impinge on nearly all adaptive fetal responses measured including growth, glucose metabolism, and insulin secretion (Davis *et al*., 2015; Macko *et al*., 2016). How these fetal responses allow normal cellular oxidation to continue, maintain viability at the expense of growth, but ultimately become maladaptive for future performance are described herein.

## Fetal metabolism and endocrinology

**Figure 6:**
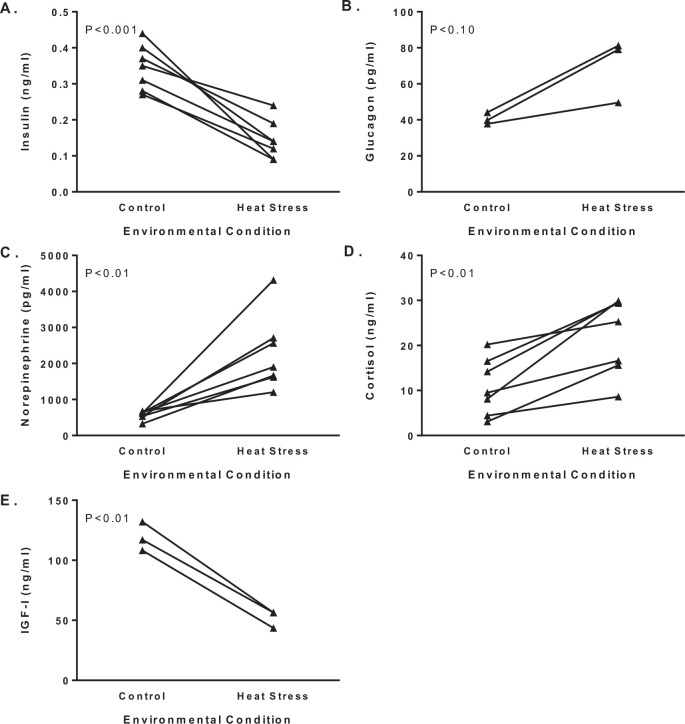
Endocrine profile in fetuses with hyperthermia-induced placental insufficiency. Mean plasma insulin (A), glucagon (B), norepinephrine (C), cortisol (D), and insulin-like growth factor I (IGF-I, E) concentrations are presented for fetuses at 90% of gestation. Group means for thermoneutral control and heat stressed ewes were reported in Limesand *et al*., 2006; Brown *et al*., 2012, 2016; Thorn *et al*., 2013; Macko *et al*., 2016; and Rozance *et al*., 2018. An ANOVA with reported study as the repeated measure identifies group (control and heat stress) differences (P-values) for hormone concentrations.

The fetus uses the umbilical uptake of nutrients to fulfill two major requirements: oxidation to fuel energy metabolism and accretion for growth and storage of substrates. Energy metabolism can be estimated from rates of oxygen consumption, which based on net umbilical oxygen uptake per fetal mass was only marginally less in IUGR fetuses compared to control fetuses (Fig. 4). For metabolic studies, the rate of oxygen consumption is one of the most useful standards of reference because the metabolic quotient defines the quantity of substrate needed to satisfy energy requirements of the fetus or fetal organs. This allows us to judge whether the quantity of nutrients being supplied to the fetus by the placenta is larger or smaller than the energy demands of the fetus, thus indicating whether the placental nutrient supply is sufficient to support fetal growth through accretion. The metabolic quotient for individual substrates are calculated as the ratio of the substrate oxygen equivalents to oxygen uptake. Oxygen equivalents are the quantity of oxygen molecules required for complete oxidation of that substrate to carbon dioxide and water. For example, when calculating the glucose/oxygen quotient, the glucose concentration difference (mmol/l) is multiplied by six and then divided by the oxygen concentration difference (mmol/l). In the fetus, the major sources for oxidative substrates are glucose, lactate, and amino acids, which explain the emphasis for studying their placental transport capacity and the resulting consequences to fetal growth when their delivery is restricted. Strikingly, the sum of the nutrient/oxygen quotients for glucose, lactate, and amino acids in IUGR fetus barely exceeds the umbilical oxygen quotient, which indicates that fetal uptake of nutrients is just sufficient to meet the oxidative requirements with no surplus for accretion (Regnault *et al*., 2013). A similar limitation in substrate availability was identified for the hindlimb quotients in IUGR fetuses (Rozance *et al*., 2018). Numerous studies have been conducted to explain how IUGR fetuses adapt to placental insufficiency by altering organ metabolism for these primary substrates as well as describing the potential endocrine regulation (Fig. 6).

Although net umbilical (fetal) glucose uptakes were not significantly lower for all reports individually, the meta-analysis performed herein for ten reports that measured umbilical glucose uptake show that glucose uptake was lower in IUGR fetuses (Fig. 4). Experiments with glucose tracers to determine rates of fetal glucose utilization and oxidation show significant alterations in glucose metabolism in IUGR fetuses with placental insufficiency (Limesand *et al*., 2007; Thorn *et al*., 2013; Brown *et al*., 2015). Body-weight specific glucose utilization rates are not different between IUGR and control fetuses, despite IUGR fetuses having markedly lower plasma insulin and glucose concentrations. In control fetuses, umbilical glucose uptake is normally equivalent to the glucose utilization rate, which demonstrates that placental glucose uptake is sufficient and glucose production is negligible. In IUGR fetuses with hyperthermia-induced placental insufficiency, glucose utilization rates exceed umbilical glucose uptake, thus the IUGR fetus has endogenous glucose production (Limesand *et al*., 2007; Thorn *et al*., 2013). The fraction of glucose oxidized to carbon dioxide is also less in IUGR fetuses, which suggests peripheral tissues may have increased glycolysis to supply lactate for hepatic glucose production (Limesand *et al*., 2007; Brown *et al*., 2015). Together, these findings identify important metabolic responses in glucose metabolism that are predicted to caused alterations in tissues such as liver and muscle, changes in endocrine factors, or a combination of both.

For glucose metabolism, three adaptations are apparent in IUGR fetuses with placental insufficiency. First, there is greater avidity for glucose uptake and utilization by fetal tissues in the presence of low glucose and insulin concentrations, indicating that there is greater insulin sensitivity in the IUGR fetus. Explanations for the greater glucose uptake capacity include a larger proportion of neuronal tissue to body weight (Fig. 5) and is supported by upregulation of glucose transporter 1 concentrations in the brain (Limesand *et al*., 2007). Furthermore, the glucose extraction efficiency and glucose uptake into both the hindlimb and myocardium are similar between control and IUGR fetuses, despite low glucose and insulin concentrations in the IUGR fetus (Barry *et al*., 2016; Rozance *et al*., 2018). Because glucose transporter expression was unaffected in IUGR muscle, adaptations in proximal insulin signaling were apparent and appear to enhance insulin sensitivity due to increased insulin receptor concentrations and decreased phosphoinositide- 3 kinase (p85) with no change in the p110 catalytic subunit (Limesand *et al*., 2007; Thorn *et al*., 2009). Second, IUGR fetuses exhibit hepatic glucose production, which is normally uncommon, but augmented by significant increases in gluconeogenic enzymes, PEPCK and glucose-6-phosphatase, perhaps in response to cAMP-response element-binding protein activation due to increases in cortisol, glucagon, and norepinephrine (Fig. 6; Limesand *et al*., 2007; Thorn *et al*., 2013). Third, enzymes that regulate the tricarboxylic acid cycle are altered in skeletal muscle and liver of IUGR fetuses. For example, pyruvate dehydrogenase kinase 4 mRNA expression is increased 5-fold in IUGR skeletal muscle. Pyruvate dehydrogenase kinase 4, when phosphorylated, inhibits pyruvate dehydrogenase, which converts pyruvate to acetyl CoA for use in the tricarboxylic acid cycle (Brown *et al*., 2015). Pyruvate carboxylase and lactate dehydrogenases expression also were depressed in the skeletal muscle from IUGR fetuses, and the former may also play a role in sparing pyruvate via glycolysis from oxidative metabolism. Interestingly, lactate output from the hindlimb was not increased in IUGR fetuses. However, the lactate/oxygen quotient was greater, which shows greater lactate output per mole of oxygen consumed by the hindlimb of the IUGR fetus (Rozance *et al*., 2018). The sum of glucose and lactate quotient was similar between control and IUGR fetuses, and the amino acid/oxygen quotient was lower. This indicates that alterations in substrate utilization are more dependent on amino acid metabolism and protein synthesis and growth are expendable.

We have followed IUGR lambs from heat stressed ewes and identified persistent augmentations in insulin sensitivity at least until two weeks of age (Camacho *et al*., 2017). These experiments demonstrate that in utero adaptations could have negative consequences during the postnatal growth period, and ultimately progress into insulin resistance because IUGR lambs with less muscle mass are adapting to their postnatal environment while also undergoing age related declines in insulin sensitivity (Gatford *et al*., 2004). Low birth weight and less muscle mass may cause metabolic limitation that decrease subsequent performance (Wu *et al*., 2006). As postnatal growth is largely dependent upon fetal growth and development, it is important to determine how management decisions could impact the growth trajectory of the fetus; however, it is not always feasible to prevent IUGR in production settings. Thus, identifying the mechanism that regulate skeletal muscle metabolism in IUGR during pre- and postnatal development is necessary.

## Pancreatic insulin secretion

Pancreatic β-cells secrete insulin in response to elevated plasma glucose concentrations to stimulate glucose uptake into fetal tissues and promote growth. In fetal sheep, β-cells become responsive to glucose after mid-gestation (Aldoretta *et al*., 1998). Since insulin cannot cross the placenta, insulin secretion from fetal pancreatic β-cells is critical for coordinating fetal growth rates with the placental glucose transport. Insulin secretion parallels changes in glucose concentrations, and both glucose and insulin concentrations are lower in IUGR fetuses (Boehmer *et al*., 2017; Fig. 6). In IUGR fetuses, glucose concentrations are chronically low (Fig. 1), which negatively affects β-cell mass and insulin secretory capacity. The IUGR fetus has impaired insulin secretion responsiveness due to two primary deficits: less β-cell mass as a consequence of slower rates of β-cell proliferation and less insulin content per β-cell (Limesand *et al*., 2005, 2006).

Another factor contributing to the suppression of insulin secretion in IUGR fetuses is persistent elevations of plasma norepinephrine and epinephrine, which inhibit insulin secretion through α2-adrenergic receptors on β-cells (Jackson *et al*., 2000). In the IUGR fetus, glucose-stimulated insulin concentrations during an adrenergic receptor blockade are equivalent to maximal insulin concentrations in control fetuses (Leos *et al*., 2010; Macko *et al*., 2013). This response occurs despite IUGR fetuses having fewer β-cells that contain less insulin. Therefore, chronic adrenergic stimulation inhibits insulin secretion from fetal β-cells, but the chronic suppression in IUGR fetuses causes developmental changes in β-cells insulin secretion responsiveness. Following chronically high norepinephrine fetal infusions, a subsequent hyper- secretory response of insulin has been confirmed in normally grown fetuses (Chen *et al*., 2014). Strikingly, in fetuses with a chronic norepinephrine infusion, the enhanced insulin secretion responsiveness to glucose and arginine persisted for five days after termination of the infusion. This is consistent with the observation of insulin hyper-secretion in week old IUGR lambs because norepinephrine concentrations are high during gestation but expected to decrease after birth when oxygen and nutrients are sufficient (Camacho *et al*., 2017; Chen *et al*., 2017; Limesand and Rozance, 2017). Surgical ablation of the fetal adrenal medulla prevents acute hypoxia-induced norepinephrine secretion and partially explains the lower glucose stimulated insulin concentrations in IUGR fetuses (Macko *et al*., 2016). While mechanisms for adrenergic inhibition of insulin secretion include distal steps in exocytosis, acute adrenergic stimulation also inhibits oxidative metabolism in β-cells and islets, which supports a role for norepinephrine to lower oxidation rates of glucose and to inhibit islet metabolism in IUGR fetuses (Kelly *et al*., 2018).

When islets from IUGR and control fetuses were analyzed for molecular changes using high throughput RNA sequencing (RNAseq), more than 1000 genes were differentially expressed and explained decreased cell proliferation (Kelly *et al*., 2017). This unbiased approach also revealed novel mechanisms underlying IUGR islet dysfunction including down regulation of immune function, suppressed Wnt signaling, adaptive stress responses, and impaired proteolysis. These transcriptional changes define adaptive responses of β-cells during IUGR and may provide the framework for understanding programming mechanisms that lead to metabolic complications in later life following hyperthermia-induced placental insufficiency.

## Skeletal muscle growth and metabolism

Lambs with fetal growth restriction are lighter at birth and grow less efficiently, yielding carcasses with insufficient muscle growth (Greenwood *et al*., 1998, 2000). In sheep, the formation of new fibers (myogenesis) is complete around 110 days of gestation after which the myofibers grow by hypertrophy, which is when declines in fiber size are detected in fetuses with placental insufficiency-induced IUGR (Maier *et al*., 1992; Wilson *et al*., 1992; Hay *et al*., 2016). Conserved myonuclear domains during early muscle growth increase protein synthesis, such that myonuclear accumulation drives growth in young animals (Pavlath *et al*., 1989). Fetal myoblast incorporation (via differentiation) into myofibers is required to increase nuclei content because nuclei within these myofibers are post-mitotic (Allen *et al*., 1979). Myoblasts proliferate and differentiate in response to an activation signal, followed by a cascade of regulatory transcription factors (e.g. Pax7, MyoD, myogenin, and others). IUGR fetuses from hyperthermia-induced placental insufficiency have similar numbers of myoblasts compared to controls but have impaired myoblast differentiation due to slower rates of myoblast proliferation. (Yates *et al*., 2014, 2016; Brown *et al*., 2017). When myoblasts are isolated and cultured from IUGR skeletal muscle, they replicate slower than controls. This is likely an intrinsic defect in the IUGR myoblast because it occurs in culture and independent of nutrient availability (Yates *et al*., 2014). Furthermore, IUGR fetuses have decreased fiber size regardless of the fiber type (Yates *et al*., 2016). These complications explain smaller muscle fibers, as well as identify programing effects that could limit growth later in life (Brown, 2014).

Skeletal muscle is a primary target for metabolic complications because it represents approximately 40% of total body weight and greater than 50% of energy expenditure (Brown, 2014). Metabolic energy requirements in skeletal muscle are met through oxidative phosphorylation. Thus, the number and efficiency of mitochondria determines metabolic capacity of muscle fibers. Traditionally, fibers are classified by oxidative capacity including Type Ia (slow oxidative), Type IIb (fast glycolytic), and Type IIa and IIx (fast oxidative; Dunlop *et al*., 2015). In the developing sheep the percentage of slow oxidative fibers (Type I) increases in number from less than 10% at 110 days gestation to greater than 25% near term and continues to increase postnatally (Maier *et al*., 1992). Myofiber area for both Type I and Type IIa fibers are decreased in hindlimb muscles from IUGR fetuses (Yates *et al*., 2016; Rozance *et al*., 2018). While there are less oxidative fibers in hindlimb muscles from IUGR fetuses, the proportion of glycolytic fibers is similar to control fetuses (Yates *et al*., 2016). We expect this developmental adaptation will lower oxidative-to- glycolytic fiber ratios and explains impaired glucose oxidation rates in the IUGR fetus (Limesand *et al*., 2007; Brown *et al*., 2015).

Long-term exposure to elevated catecholamines down regulates adrenergic receptor concentrations, lowers sensitivity, and impairs skeletal muscle metabolism (Yates *et al*., 2014). Chronic adrenergic stimulation is associated with adaptive programming responses in fetal metabolic tissues: pancreatic islets (Chen *et al*., 2014; Camacho *et al*., 2017), skeletal muscle (Yates *et al*., 2012), and adipose (Chen *et al*., 2010). Moreover, adaptations to chronic adrenergic stimulation persist after the insult, creating the potential for life-long metabolic programming. Specifically, adrenergic receptor β2 mRNA concentrations are reduced >60% in IUGR fetuses and lambs but adrenergic receptors β1 and β3 are not different (Chen *et al*., 2010). Therefore, potential desensitization of adrenergic receptor β2, but not other β-adrenergic receptors, might impair insulin responsiveness in skeletal muscle because it persists in lambs after birth.

## Cardiac metabolism

The effects of maternal heat stress on fetal development and maturation are also evident in the heart. Blood flow to essential fetal organs (the brain, heart, and adrenal glands) is increased preferentially in response to acute bouts of hypoxia. As a result, blood flow decreases to the gastrointestinal, renal, and peripheral vasculature. This pattern for redistribution of cardiac output is also maintained during chronic periods of hypoxia such as those found in hyperthermia-induced placental insufficiency, which may result in the asymmetric fetal growth (Fig. 5).

IUGR fetal myocardium responds through unique metabolic and cardiovascular adaptations that support myocardial growth and function. The reduced circulating anabolic factors (Fig. 6) present in IUGR would be expected to suppress cardiac growth, as the fetal heart is sensitive to insulin and IGF-1, and reliant on glucose and lactate as major carbon sources for metabolism (Bartelds *et al*., 1998, 2000). The myocardium of the IUGR fetus responds by increasing plasma membrane concentrations of the insulin- stimulated glucose transporter 4 and insulin receptor β protein (Barry *et al*., 2006). In contrast, myocardial membrane protein concentrations of glucose transporter 1 are unchanged in the IUGR fetus. Additionally, blood flow to, and glucose delivery/uptake by, the left ventricle is significantly increased by insulin stimulation (Barry *et al*., 2016). These adaptations appear to promote myocardial energy supply and utilization by increasing its sensitivity to insulin, which supports cardiac growth despite the significant nutrient deprivation.

Fetal cardiac function appears to be relatively unaffected by placental insufficiency because heart rates are similar between IUGR and control fetuses (Galan *et al*., 2005; Barry *et al*., 2016). However, IUGR fetuses have increased indices of umbilical artery resistance and a significant reduction in umbilical blood flow, which did not always cause greater mean aortic blood pressure (Galan *et al*., 2005; Barry *et al*., 2016). The increased placental vascular resistance might reflect a mechanism by which the fetus is able to increase extraction of nutrients from the placenta as discussed above. A similar model that induces IUGR by removing the majority of the uterine caruncles in the sheep also found no difference in fetal blood pressure under baseline conditions; however, there was a greater hypotensive effect in IUGR fetuses following administration of phentolamine, an α-adrenergic antagonist, and captopril, an angiotensin-converting enzyme inhibitor (Edwards *et al*., 1999; Danielson *et al*., 2005). These findings indicate that the α-adrenergic and renin-angiotensin systems have a greater role in blood pressure maintenance in IUGR fetuses, which are likely involved in mediating some of the organ sparing phenomena present in this model (McMillen *et al*., 2001; Danielson *et al*., 2005).

## Conclusions

We have presented evidence for prolonged exposure to heat stress causing placental insufficiency in ruminants. Maladaptive responses during development, which include fetal growth restriction, persist as lifelong deficiencies lowering the performance and health of the animal. We discuss how the enlarged transplacental gradient for oxygen and glucose facilitates umbilical uptakes but results in low blood oxygen and plasma glucose concentration in the fetus. These conditions slow growth by altering glucose metabolism, decreasing amino acid clearance, and decreasing anabolic hormones while increasing catabolic hormones.
